# Microwave assisted catalytic co-pyrolysis of banana peels and polypropylene: experimentation and machine learning optimization

**DOI:** 10.1039/d5ra03913d

**Published:** 2025-08-11

**Authors:** Nilesh S. Rajpurohit, Shruti Sinha, Ramesh Potnuri, Chinta Sankar Rao, Harshini Dasari

**Affiliations:** a Control Systems & Machine Learning Research Laboratory, Department of Chemical Engineering, National Institute of Technology Karnataka Surathkal-575025 India csrao@nitk.edu.in; b Chemical Engineering Department, Manipal Institute of Technology Manipal Academy of Higher Education Manipal Udupi Karnataka 576104 India harshini.dasari@manipal.edu

## Abstract

The growing accumulation of agricultural and plastic waste poses serious environmental challenges, necessitating sustainable and efficient valorization strategies. This study investigates the microwave-assisted catalytic co-pyrolysis of banana peels and polypropylene, using graphite as a susceptor and potassium hydroxide as a catalyst. Experiments were conducted by varying biomass and plastic quantities and microwave power levels to study their effects on product yields and thermal performance. The process effectively converted waste materials into valuable products, with oil yield increasing with microwave power and optimized biomass-to-plastic ratios. The rate of mass loss and heating rate were found to significantly influence overall conversion efficiency. A support vector regression (SVR) model was developed to predict yields based on input parameters, achieving a coefficient of determination ranging from 0.81 to 0.99, which demonstrates the reliability of machine learning in capturing complex thermochemical behavior. 3D plots illustrated the nonlinear effects of process variables on yields. Fourier Transform Infrared Spectroscopy (FTIR) and X-ray Diffraction (XRD) analyses of char confirmed functional groups and crystalline phases, suggesting its suitability for applications like adsorbents or catalysts. Brunauer–Emmett–Teller (BET) analysis showed multilayer adsorption, while thermogravimetric analysis (TGA) highlighted distinct thermal degradation patterns of the feedstocks. These results affirm the promise of integrating experiments with ML for efficient waste-to-energy conversion.

## Abstract 2

The growing accumulation of agricultural and plastic waste poses serious environmental challenges, necessitating sustainable and efficient valorization strategies.

## Graphical abstract

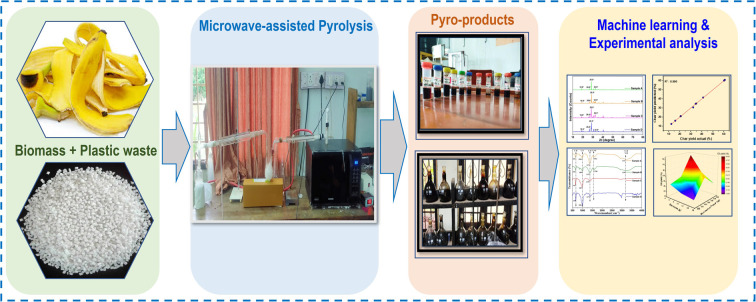

## 1 Introduction

When compared to non-renewable energy sources, biomass as a renewable energy source has several benefits. Biomass is derived from biological elements that can quickly regenerate or refill, unlike fossil fuels like coal, oil, and natural gas.^1^ Biomass becomes a sustainable and environmentally responsible energy source due to this replenishing cycle.^2^ Non-renewable energy sources, in contrast, are limited and gradually decrease, raising challenging questions about energy security and driving up costs.^3^ Biomass combustion is considered nearly carbon-neutral, as the CO_2_ emitted is largely offset by that absorbed during growth. In contrast, non-renewable energy sources generate substantial CO_2_ emissions, contributing to climate change.^4^ Biomass converts forestry and agricultural waste into useful energy, reducing waste and offering a sustainable alternative to non-renewable sources with lower environmental impact.^5^

With diminishing fossil fuel availability, researchers are exploring thermochemical routes to convert biomass waste into hydrocarbon fuels. Key methods include gasification, liquefaction, combustion, and pyrolysis.^6^ Among these, pyrolysis has received particular attention for its effectiveness in processing various biomass sources such as forestry residues, agricultural waste, and food industry by-products.^7^ Pyrolysis is a thermochemical process that involves the thermal decomposition of carbon-rich solid materials under an inert atmosphere, typically using gases such as nitrogen, argon, or helium. This method is widely employed for converting various types of waste, ranging from biomass and plastics to electronic waste, sewage sludge, and used tires, into valuable products.^8^ The technique is recognized for its rapid reaction kinetics, high conversion rates, and operational adaptability.^9^ Co-pyrolysis, a variation of this process, offers several benefits compared to traditional pyrolysis. Notably, it enables the simultaneous processing of different feedstocks, such as blending biomass with plastic, rubber, or other waste materials, leading to synergistic interactions that can improve both the yield and quality of the end products.^10^ This approach also broadens the scope for valorizing heterogeneous and otherwise challenging waste streams, thus enhancing material recovery and reducing environmental burden.^11^

Co-pyrolysis offers feedstock flexibility and process optimization, while catalysts enhance efficiency and selectivity by reducing unwanted byproducts.^12^ This makes it a promising approach for sustainable waste management and circular economy development.^13^ Microwave-assisted co-pyrolysis offers several advantages over conventional methods, including reduced reaction time,^14^ rapid and uniform heating,^15^ and improved control over temperature and heat distribution, enhancing product selectivity and minimizing byproducts.^16^ It also improves energy efficiency by directly heating feedstocks^17^ and facilitates better moisture removal, enhancing product quality.^18,19^ Microwave-assisted co-pyrolysis offers the flexibility to process a broad spectrum of biomass and plastic waste as feedstocks. The co-processing of biomass and plastics not only facilitates the simultaneous valorization of renewable and synthetic waste materials but also enhances overall waste management efficiency. This synergistic approach supports both environmental sustainability and resource recovery. In microwave-assisted systems, susceptors that efficiently absorb microwave energy are often incorporated to improve thermal distribution and promote uniform, localized heating within the reaction medium, thereby optimizing the pyrolytic conversion process.^20^ These susceptor materials have high dielectric properties, allowing them to absorb microwaves and convert them into heat. Examples of commonly used susceptor materials include carbonaceous materials like activated carbon, graphite, or carbon black.^21^ Potassium hydroxide (KOH) has been widely identified as an effective catalyst in microwave-assisted co-pyrolysis processes, offering significant improvements in both conversion efficiency and product yield.^22^ Its catalytic role is primarily attributed to its ability to enhance cracking and reforming reactions, thereby facilitating the breakdown of complex organic structures present in biomass and plastic feedstocks. Through these mechanisms, KOH promotes the formation of valuable gaseous, liquid, and solid products, contributing to the overall efficiency and selectivity of the pyrolytic process.^23^

For modeling and optimizing the complicated processes involved in biomass conversion, machine learning (ML) approaches provide useful tools. First, predictive models based on historical data and test results can be created using ML algorithms.^24^ The relationships between different input parameters, such as biomass composition, reaction conditions, and catalyst qualities, and the resulting yields of desired products can be captured by these models. Large datasets can be analyzed by ML to find patterns and connections that would not be visible using more conventional analytical techniques.^25^ This makes it possible for researchers to forecast yields with greater accuracy and to optimize process variables for optimal effectiveness.^23^ These algorithms are capable of modifying and optimizing reaction conditions in response to changing factors by continually observing and examining real-time data from the thermochemical system. Higher yields and less energy use are the results of this adaptive control's contribution to the maintenance of ideal conditions and improved overall performance.^26^

This study investigates the microwave-assisted co-pyrolysis of banana peel powder, a low-cost renewable agro waste, and polypropylene, a widely used plastic contributing to environmental pollution. Although co-pyrolysis has attracted considerable attention as a sustainable waste management strategy, limited studies have explored the combined valorization of fruit waste and synthetic polymers using microwave energy, particularly through integrating experimental techniques with machine learning based optimization. To address this research gap, the present work examines the influence of varying feedstock quantities and microwave power levels on process efficiency and product yields. Graphite and potassium hydroxide were used as a susceptor and a catalyst, respectively, to improve microwave absorption and catalytic reactivity. A design of experiments approach was employed to assess the combined effects of biomass-to-plastic ratios and operating power on the distribution of pyrolysis products. The solid, liquid, and gaseous products were carefully collected and analyzed. Key process parameters, including average heating rate, susceptor thermal efficiency, mass loss rate, and conversion, were evaluated using machine learning algorithms to identify optimal operating conditions. The solid char product was further characterized using Brunauer–Emmett–Teller surface area analysis, X-ray diffraction, and Fourier transform infrared spectroscopy to determine its structural and functional properties. This integrated approach offers new insights into the efficient utilization of organic and plastic waste through microwave-assisted co-pyrolysis supported by predictive modeling and material characterization.

## 2 Experimental section

### 2.1 Materials and methods

**Fig. 1:**
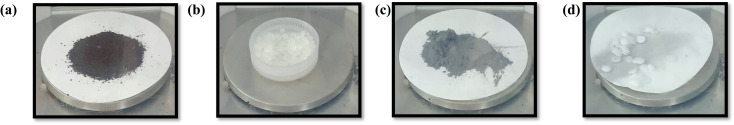
(a) Banana peels powder biomass, (b) polypropylene plastic, (c) graphite susceptor, (d) KOH catalyst.

Banana peels were procured from a local vendor in Surathkal, Karnataka, India. The peels were cut into smaller pieces and dried in a hot air oven at 120 °C to remove moisture. The dried material was then ground and sieved to obtain banana peel powder with particle sizes ranging from 0.2 mm to 1 mm. Graphite powder with a particle size of 150 μm was utilized as the microwave susceptor to enhance heat absorption. Potassium hydroxide pellets, initially around 100 μm in diameter, were pulverized into fine powder before being used as a catalyst. Polypropylene, used as the plastic feedstock, was obtained directly from a commercial supplier (Otto Biochemicals). The microwave-assisted co-pyrolysis experiments employed banana peel powder and polypropylene as feedstocks, graphite as the susceptor, and potassium hydroxide as the catalyst. Fig. 1 shows the biomass, plastic, susceptor, and catalyst used in the experiment. The feed mixture, along with the susceptor, was placed in a flat-bottomed borosilicate glass flask, which functioned as the pyrolysis reactor. This reactor was positioned inside a ceramic wool-insulated muffle to minimize heat loss, and Teflon® tape was used to secure the insulation and provide structural stability to the setup.

### 2.2 Experimental setup and procedure

**Fig. 2:**
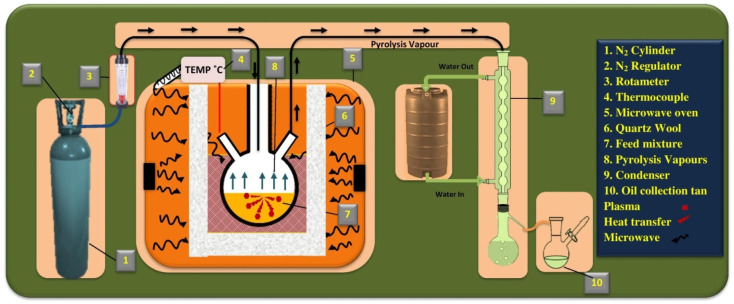
The experimental setup for the microwave assisted copyrolysis.

The pyrolysis experiments were conducted in a microwave oven with a 23-L capacity (Make: Samsung MS23A). A circular opening was created on the upper part of the oven to facilitate the integration of the thermocouple, gas purging line, and condenser with the borosilicate flask. The feedstock (biomass and plastic), catalyst, and susceptor mixture were maintained in a single-neck, round-bottom flask with a volume capacity of 500 mL. During the pyrolysis process, the temperature of the sample bed was monitored using a microwave-compatible K-type thermocouple. To maintain thermal stability within the oven, the borosilicate flask was encased in ceramic wool insulation. Two water-cooled condensers were used in series to condense the vapors produced during pyrolysis and collect the condensate as oil. The experimental setup for the microwave-assisted pyrolysis is shown in Fig. 2.

A total of 13 sets of experiments were performed with biomass and plastic of varying weights of 5 g, 10 g, and 15 g, respectively. Also, microwave power was varied to 300 W, 450 W, and 600 W along the runs. Graphite susceptor and KOH catalyst were used in a fixed amount of 5 g each during the run. Microwave-assisted pyrolysis experiment was conducted for a total time of 10 min each. During the experiment, the temperature was noted at an interval of 30 seconds, and the average heating rate was calculated from it. After the experiment, the setup is allowed to cool. The initial weight of the setup and the final weight are noted down. In each experimental run, the residual solid remaining in the reactor, comprising both char and graphite, was quantified using an analytical balance. The net char yield was determined by deducting the initial mass of graphite from the total solid residue. The mass of bio-oil recovered post-condensation, consisting of both aqueous and organic components, was also measured. To isolate the organic constituents in the aqueous phase of the bio-oil, a solvent extraction was performed using dichloromethane. The quantity of non-condensable gases was estimated through a mass balance approach. Table 1 outlines the specific experimental parameters, including reaction duration (mins), biomass and plastic feedstock weights (g), applied microwave power (W), quantities of susceptor and catalyst (g), heating rate (°C min^−1^), and the resulting product yields char, gas, and oil along with overall conversion efficiency, mass loss rate, and susceptor thermal energy.

**Table 1:** Experimental data for microwave assisted co pyrolysis of banana peels biomass and polypropylene plastic^a^

Expt. run	BP (g)	PP (g)	MP (W)	HR (°C min^−1^)	Char yield (%)	Oil yield (%)	Gas yield (%)	Conv. (%)	Rate of mass loss (wt% min^−1^)	Susceptor thermal energy (J g^−1^)
1	5	5	450	39.2	32.2	35.7	32.0	67.8	6.7	465.7
2	15	5	450	50.4	35.0	33.8	31.1	64.9	6.5	631.5
3	5	15	450	45.3	37.7	26.6	35.7	62.30	6.2	556.3
4	15	15	450	45.7	23.6	37.9	38.3	76.3	7.6	561.8
5	5	10	300	44.8	44.7	27.1	28.1	55.2	5.5	526.9
6	15	10	300	32.8	60.1	20.3	19.4	39.8	3.9	376.3
7	5	10	600	55.3	15.9	50.2	33.8	84.0	8.4	687.0
8	15	10	600	66.0	20.7	27.0	52.2	79.2	7.9	880.2
9	10	5	300	37.6	41.2	26.4	32.3	58.7	5.8	432.5
10	10	15	300	28.3	60.9	18.0	20.9	39.0	3.9	315.6
11	10	5	600	59.2	12.4	31.2	56.4	87.6	8.7	751.0
12	10	15	600	54.4	21.0	38.7	40.2	79.0	7.9	691.0
13	10	10	450	61.2	34.4	22.4	43.0	65.5	6.5	775.5

a BP: banana peel, PP: polypropylene, MP: microwave power, HR: heating rate.

### 2.3 Energy balance calculations

The design of large scale pyrolysis systems requires careful consideration of thermal and microwave energy demands to ensure process efficiency and scalability. In evaluating the energy balance, multiple factors were accounted for, including conductive heat losses, the input of microwave energy, the sensible heat retained by the char, susceptor, and borosilicate reactor components, the effective microwave energy output, and the thermal energy utilized for the decomposition of the feedstock. These parameters collectively influence the overall energy efficiency and feasibility of microwave-assisted pyrolysis at industrial scales. The following eqn (1) can calculate the microwave energy requirement.

1 *E*_m_ = *E*_p_ × *t*

Here, *E*_m_ represents the total microwave energy consumed during the process, while *E*_p_ denotes the applied microwave power. The variable *t* corresponds to the duration of the pyrolysis operation. The sensible heat retained by the graphite susceptor and the borosilicate reactor flask was estimated using eqn (2) and (3), respectively.

2 *Q*_susceptor_ = *m*_s_ × *C*_ps_ × d*T*

3 *Q*_borosilicate_ = *m*_B_ × *C*_pB_ × d*T*

where, *Q*_susceptor_ and *Q*_borosilicate_ refer to the sensible heat stored in the char susceptor powder and the borosilicate flask, respectively. *m*_S_ and *m*_B_ correspond to the mass of the char susceptor and the borosilicate flask. The specific heat capacities of the char susceptor and the borosilicate material were determined using eqn (4) and (5).4
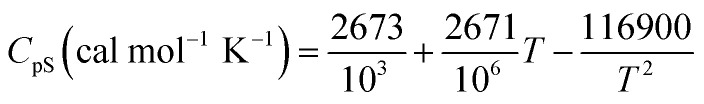
5
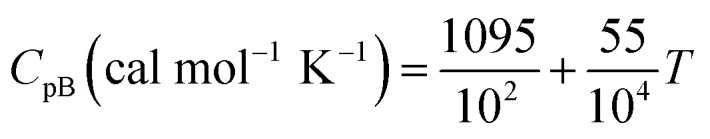
where *C*_pS_ and *C*_pB_ represent the specific heat capacities of the graphite susceptor and the borosilicate flask, respectively, reflecting their ability to store thermal energy per unit mass under constant pressure conditions. Susceptor thermal energy (J g^−1^) is calculated using the following eqn (6)6
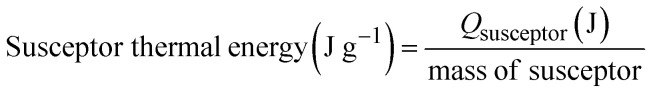


### 2.4 Support vector regression machine learning model

Support Vector Regression (SVR) is based on the principles of Support Vector Machines (SVM), which enable the modeling of non-linear relationships by mapping input features to a higher-dimensional space. The primary objective of SVR is to identify a hyperplane that best fits the data while maintaining deviations within a predefined margin, known as the epsilon tube.^27^ The data points lying within or on the boundary of this tube, referred to as support vectors, play a critical role in defining the model.^28^ The mathematical basis for the SVR is given in the SI. In this study, an SVR model was developed with three input variables: biomass weight, plastic weight, and microwave power. Further, standard scaling was performed on the input variables to bring them to the same scale and improve the model's prediction. In machine learning, standard scaling, commonly referred to as *z*-score normalization, is a common method for preparing data. It entails changing the feature values of a dataset to have unit variance and a zero mean. Standard scaling aims to scale all features similarly, which is advantageous for some machine learning methods. Standard scaling entails dividing by the standard deviation after subtraction each feature's mean from its values. Each feature will have a mean of zero and a standard deviation of one thanks to this change. The usual scaling formula is as follows:

7 *z* = (*x* − *μ*)/*σ*

where *z* represents the standardized value, *x* is the original feature value, *μ* is the mean of the feature, and *σ* is the standard deviation of the feature.

Hyperparameter tuning for the SVR model was performed using the GridSearchCV method, which conducts an exhaustive search over a predefined grid of hyperparameters to identify the optimal combination.^29^ Common SVR hyperparameters include the regularization parameter (*C*), epsilon-tube width (*ε*), and Kernel type. GridSearchCV evaluates each combination using K-fold cross-validation, where the dataset is split into K folds: the model is trained on K-1 folds and validated on the remaining one, rotating this process K times. The average performance metric (*e.g.*, mean squared error) across all folds determines the best configuration. The optimal model and corresponding hyperparameters are accessible *via* the best_estimator_ and best_params_ attributes of the GridSearchCV object.^30^ This approach enables systematic and robust tuning of SVR hyperparameters, improving the model's generalization to unseen data.^31^ The model is evaluated based on the coefficient of determination (*R*^2^)8
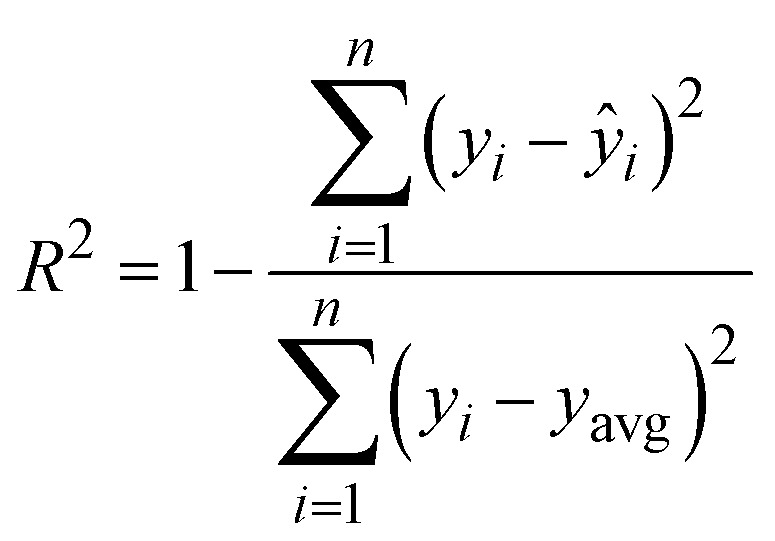
where *ŷ*_*i*_, *y*_*i*_, and *y*_avg_ are predicted output value, actual output value, and an average of actual output values, respectively, and *n* is the number of samples. The model is said to be more precise if *R*^2^ is close to 1. The SVR model is chosen over other models such as Random Forest Regression, Artificial Neural Networks, and Gradient Boosted Trees. These models were initially tested; however, due to the limited size of the experimental dataset, they demonstrated inferior performance and signs of overfitting. SVR, being more suitable for small datasets with non-linear behavior, showed better generalization capability and prediction accuracy.

The steps involved in SVR modeling are given as

1. Data collection

Experimental data from microwave-assisted co-pyrolysis of BP and PP were generated in the laboratory.

2. Data preprocessing

Features (biomass weight, plastic weight, and microwave power) and target variable (*e.g.*, oil yield, char yield, gas yield, *etc.*) were extracted.

Feature scaling was applied using StandardScaler to normalize the data.

3. Model selection

Support Vector Regression (SVR) was selected based on its suitability for small datasets and its robustness in handling nonlinear regression problems.

4. Train-test split

The dataset was split into 80% training and 20% testing using a fixed random seed to ensure reproducibility.

5. Hyperparameter optimization

A GridSearchCV method with 4-fold cross-validation was used to identify the optimal values of SVR parameters:

i. Kernel function: rbf

ii. Regularization parameter (*C*)

iii. Kernel coefficient (gamma)

6. Model training

The optimized SVR model was trained using the scaled training dataset.

7. Model evaluation

The model performance was evaluated using the following metrics

i. *R*^2^ (Co-efficient of Determination)

ii. MAE (Mean Absolute Error)

iii. MAPE (Mean Absolute Percentage Error)

iv. RMSE (Root Mean Squared Error)

8. Visualization

A parity plot (actual *vs.* predicted values) was generated to visualize model accuracy and agreement.

Model results were analyzed to understand prediction trends using 3D-surface plots.

## 3 Results and discussion

### 3.1 Feedstock characterization

Table 2 shows the proximate and elemental composition of the feedstocks, reported on a dry basis. It is observed that banana peels have a moisture content of 0.49%. Banana peels have a high volatile matter content of 71.5 wt%, fixed carbon content is around 23.1 wt% and ash is 4.7 wt%. Fixed carbon (FC) represents the carbonaceous portion of the biomass that remains after volatile matter (VM) and moisture has been driven off during combustion. In this case, banana peels have relatively low fixed carbon content, indicating that they may have limited energy content compared to other biomass sources with higher fixed carbon content. The high volatile matter content suggests that banana peels would be readily combustible and could be used for thermal energy generation. The low ash content is desirable for biomass applications, as high ash content can lead to increased slagging and fouling during combustion. Banana peels' low ash content indicates their potential suitability for combustion-based applications. The low moisture content is advantageous for energy applications, as moisture contributes to the energy required for drying the biomass before combustion.^32^

**Table 2:** Ultimate analysis and proximate analysis of banana peels and polypropylene^a^

Sr. no.		Ultimate analysis (wt%)					Proximate analysis (wt%)			
		N	C	H	S	O	FC	VM	Ash	MC
1	Banana peels	1.2	44.8	8.4	0	45.3	23.1	71.5	4.7	0
2	Polypropylene	0	84.6	7.0	0	8.3	5.3	93.7	0	1

a M-moisture, VM-volatile matter, FC-fixed carbon, A-ash content, C-carbon, H-hydrogen, N-nitrogen, S-sulphur, O-oxygen.

The ultimate analysis of banana peels shows that it has a high amount of carbon content at 44.87 wt% and very low nitrogen content at 1.28 wt%. Sulphur content in biomass is not detected and biomass has around 45.38% oxygen content. Nitrogen (N) is a vital element in biomass, as it contributes to the protein content. The relatively low nitrogen content in banana peels indicates limited protein content, consistent with the biomass's non-proteinaceous nature. The high carbon and oxygen content suggests that banana peels primarily consist of carbohydrates, cellulose, and hemicellulose, which are typical constituents of plant material.^33^

For polypropylene, it is observed that the carbon content is very high (84.6 wt%) and the hydrogen content is 7.03 wt%. % with no detection of nitrogen and sulfur content. The absence of detectable nitrogen and sulfur indicates that polypropylene is primarily composed of carbon, hydrogen, and oxygen. This composition is consistent with the elemental makeup of most hydrocarbon-based polymers.^34^ Proximate analysis of polypropylene gives a higher amount of VM present with very little amount of moisture content (MC) and FC. Polypropylene's high VM content indicates that it is highly combustible and can release a substantial amount of energy when burned. The absence of ash content suggests that polypropylene leaves behind virtually no residue upon combustion, which can be advantageous in applications where minimal ash production is desired, such as in certain industrial processes. The low MC is favorable for applications involving combustion or heat treatment.^35^

### 3.2 Thermogravimetric Analysis (TGA)

**Fig. 3:**
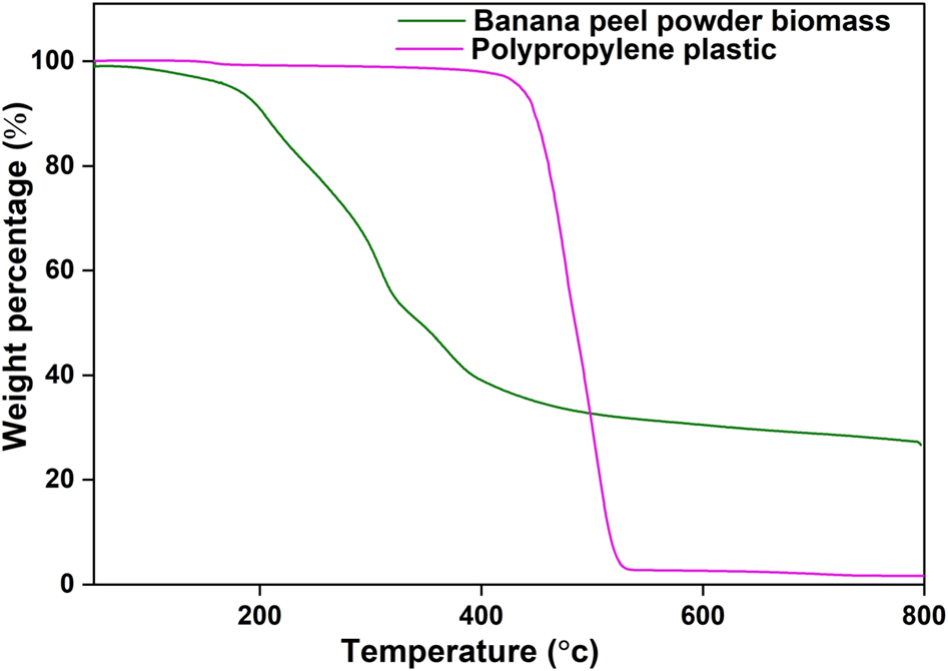
TGA analysis of banana peels biomass and polypropylene plastic.

Thermogravimetric analysis, often referred to as TGA, is a vital analytical technique employed across a wide spectrum of scientific and industrial disciplines. This technique serves as a cornerstone in the investigation of materials' thermal behavior, providing insights into their decomposition, sublimation, phase transitions, and overall thermal stability. A biomass sample of weight 8.2 mg was used for analysis with a heating rate of 50 °C min^−1^. The sample was heated at temperatures from 50 °C to 900 °C at 50 °C min^−1^. TGA analysis for banana peel powder is shown in Fig. 3. It can be observed that around 500 °C, there is a mass loss of around 65% and 23.03% of the initial weight of the sample is left at the end of the experiment. The initial mass loss in the TGA curve is often attributed to the removal of moisture or water content from the banana peel biomass.^36^ This is a common feature in TGA curves for many organic materials. The moisture content evaporates quickly when exposed to elevated temperatures, resulting in an abrupt drop in mass. Cellulose is one of the primary components of plant-based biomass, including banana peels.^37^ The sudden mass loss might also correspond to the thermal degradation of cellulose, which typically occurs in a distinct temperature range. This decomposition process can involve the cleavage of glycosidic bonds within the cellulose structure. Hemicellulose is another component of plant biomass that decomposes upon heating. The sudden mass loss could be associated with the decomposition of hemicellulose, which typically occurs at lower temperatures than cellulose.^38^

Similarly, Fig. 3 shows the TGA analysis of polypropylene plastic. A plastic sample of 22.4 mg was used, which was heated from 50 °C to 900 °C at a heating rate of 50 °C min^−1^. It can be observed that there is a sudden loss in the weight of the sample in the temperature range of 425 to 520 °C, where around 95 wt% of the weight is lost. Polypropylene is a thermoplastic polymer composed of repeating propylene units. When subjected to elevated temperatures, thermoplastic polymers typically undergo thermal degradation, which involves the breaking of chemical bonds within the polymer chains.^39^ The fact that around 95% of the weight is lost in this temperature range indicates that the thermal decomposition of polypropylene is quite extensive. During this process, the polymer chains break down into smaller fragments, resulting in the release of volatile products, such as hydrocarbons and gases, which are responsible for the mass loss.^39^ After the significant mass loss in this temperature range, there is typically a residue left behind. This residue may contain char or non-volatile compounds that are more thermally stable than the original polypropylene.

### 3.3 Predictions of pyro-product yields

#### 3.3.1 Oil yield

**Fig. 4:**
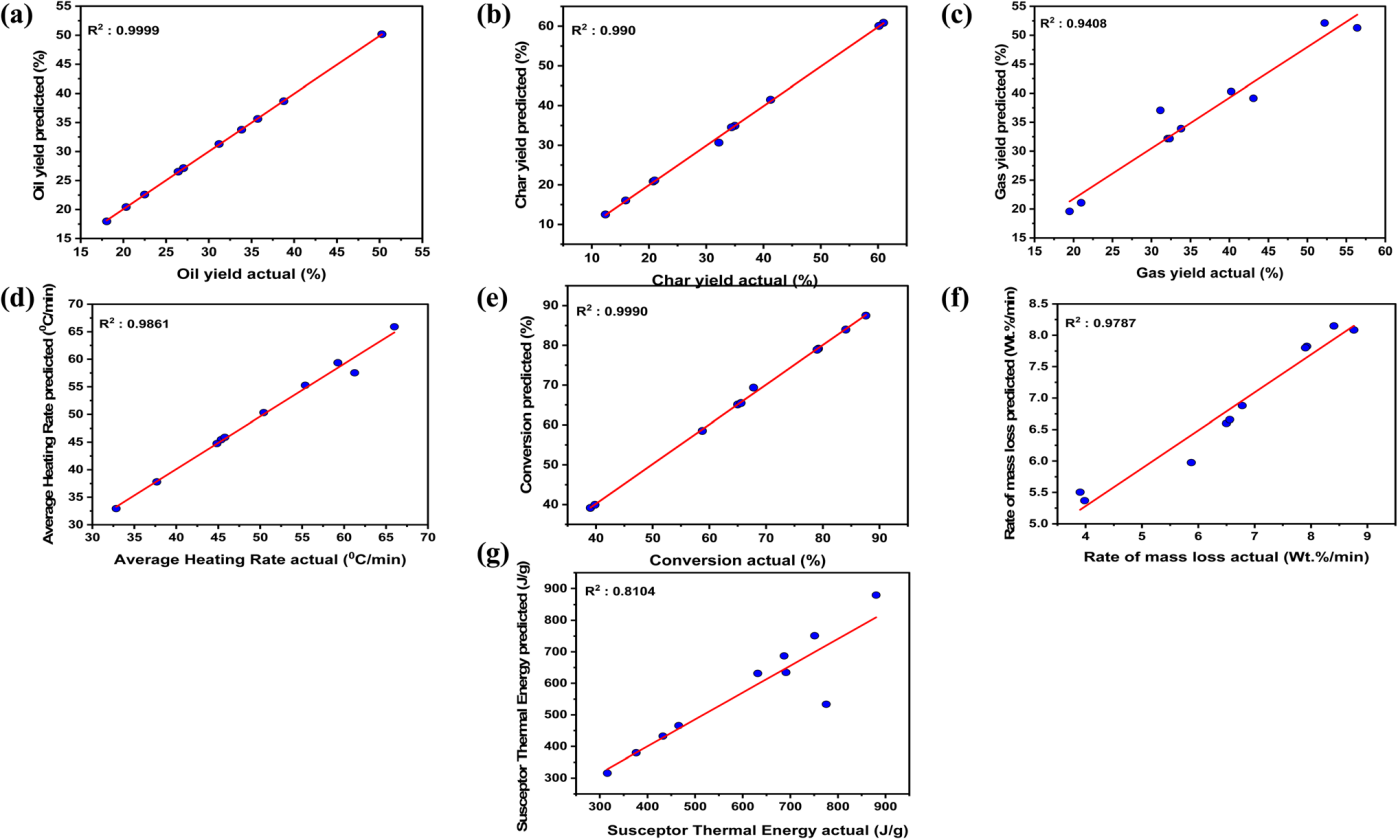
Parity plots of (a) oil yield, (b) char yield, (c) gas yield, (d) avg. heating rate, (e) conversion, (f) rate of mass loss, (g) susceptor thermal energy (%).

**Fig. 5:**
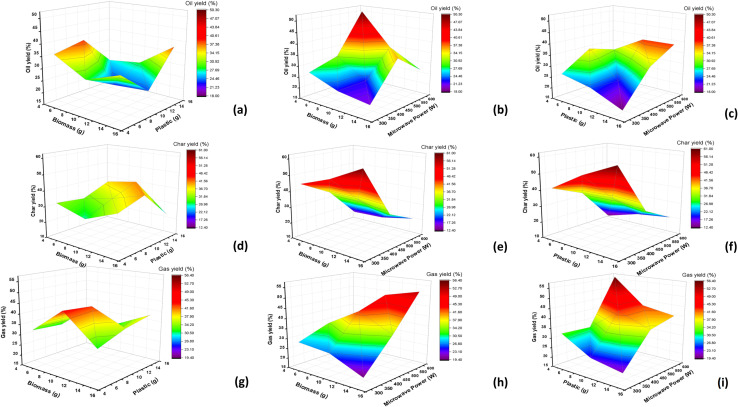
3D surface plots of (a) oil yield with biomass and plastic, (b) with biomass and microwave power, (c) with plastic and microwave power, (d) Char yield with biomass and plastic, (e) with biomass and microwave power, (f) with plastic and microwave power, (g) gas yield with biomass and plastic, (h) with biomass and microwave power, (i) with plastic and microwave power.

The SVR model was developed using the GridSearchCV method from the experimental data collected. To determine the prediction of the model, *R*^2^ plots were generated with the *x*-axis representing the actual oil yield values from the experimental data and the *y*-axis representing the predicted oil yield values by the machine learning model. Fig. 4(a) shows the *R*^2^ plots for oil yield prediction. It can be observed that the SVR model developed was able to predict the oil yield with higher accuracy, giving an *R*^2^ value of 0.99. The error metrics obtained are given in Table S1 (SI). Fig. 5(a) shows 3-D plots for oil yield variation with changes in biomass quantity and plastic quantity. It can be observed that a higher amount of biomass, while keeping plastic quantity low, leads to an overall decrease in oil yield. Increasing the amount of banana peel biomass while keeping the plastic quantity low can result in a higher ratio of biomass to plastic. This higher biomass-to-plastic ratio may dilute the heating and catalytic properties of the plastic. As a result, the overall efficiency of the pyrolysis process may decrease, leading to a lower oil yield. With a lower quantity of plastic, there might be insufficient heat generation in the system. Polypropylene plastic is known to absorb microwave energy efficiently and convert it into heat, promoting pyrolysis reactions. When the plastic quantity is low, the amount of heat generated may not be sufficient to drive the pyrolysis process effectively, leading to a decrease in oil yield.^40^

Also, it is observed that increasing the amount of plastic while keeping biomass quantity low leads to an overall increase in oil yield. Polypropylene plastic has good dielectric properties, meaning it can efficiently absorb microwave energy and convert it into heat. By increasing the amount of plastic, more microwave energy is absorbed, resulting in higher temperatures within the system. This enhanced heating efficiency can promote the pyrolysis of both the plastic and the banana peel biomass, leading to increased oil yield. The combination of polypropylene plastic and banana peel biomass in microwave co-pyrolysis can create synergistic effects. The plastic acts as a heating source, providing additional thermal energy to the system. This extra heat can help in the decomposition of the biomass, releasing more volatile compounds, including oil. The interaction between the plastic and the biomass can enhance the overall pyrolysis process, leading to a higher oil yield compared to using biomass alone. Also, higher amount of both biomass and plastic leads to higher oil yield because when higher amounts of biomass and plastic are present, there is an increased surface area for energy absorption, resulting in higher heat generation within the system. This elevated temperature facilitates the pyrolysis process and enhances the release of oil from the biomass.^41^

Fig. 5(b) shows 3-D plots for oil yield variation with changes in biomass quantity and microwave power. It can be observed that with low biomass quantity, when microwave power is increased, it leads to an increase in oil yield. Increasing the microwave power results in higher energy input into the system. This increased energy generates higher temperatures within the reactor. With low biomass quantity, there is less material to absorb the heat, allowing more energy to be concentrated on the available biomass. The higher temperatures facilitate more efficient pyrolysis reactions, leading to an increased oil yield. Also, with higher biomass quantity, when microwave power is increased it leads to an increase in oil yield till 450 W and then there is a decrease in oil yield till 600 W power. With higher biomass quantity, more material needs to be heated to achieve the desired pyrolysis temperature. Increasing the microwave power can initially improve the heating efficiency and accelerate the heating process. This leads to a higher oil yield as the biomass reaches the optimal temperature for co-pyrolysis. However, at higher microwave power levels beyond 450 W, the residence time might be insufficient for complete pyrolysis. This can result in incomplete conversion of the biomass and a subsequent decrease in oil yield. As the biomass quantity increases, the heat transfer within the reactor can become less efficient. The larger biomass mass may impede the uniform distribution of heat, resulting in temperature gradients and localized overheating. This uneven heat distribution can lead to incomplete pyrolysis and reduced oil yield. At higher microwave power levels, the heat transfer limitations may become more pronounced, causing a decrease in oil yield.^42^

Fig. 5(c) shows 3-D plots for oil yield variation with changes in plastic quantity and microwave power. Here, as microwave power is increased with low plastic quantity, oil yield is also increased. The microwave power increases, resulting in higher energy input into the system. This increased energy generates higher temperatures within the reactor, promoting more efficient pyrolysis reactions. With a low PP plastic quantity, more of the microwave energy is absorbed by the biomass, leading to higher temperatures within the biomass and enhanced thermal decomposition. The higher temperatures facilitate the release of oil from the biomass, resulting in an increased oil yield. Higher microwave power levels result in more rapid heating of the biomass. This rapid heating increases the reaction rates within the biomass, accelerating the decomposition of organic compounds and the release of oil. The higher reaction rates contribute to an increased oil yield in microwave co-pyrolysis.^43^ Increasing microwave power from 300 W to 600 W with a higher amount of PP plastic leads to a significant increase in oil yield. Increasing the microwave power results in higher energy input into the system, leading to increased heating. With a higher amount of PP plastic, there is a greater capacity to absorb and convert microwave energy into heat. This enhanced heating raises the temperature within the reactor, promoting more efficient pyrolysis reactions. The higher temperatures facilitate the thermal decomposition of the biomass, resulting in an increased oil yield.

#### 3.3.2 Char yield

Similarly, the SVR model developed for the experimental data was used to predict the char yield. Fig. 4(b) shows an *R*^2^ plot for char yield prediction for microwave-assisted co-pyrolysis of biomass and plastic, with actual experimental values on the *x*-axis and predicted char yield values on the *y*-axis. It is observed that the model was able to fit in line with the experimental results with an *R*^2^ value of 0.99. It is observed in Fig. 5(d), which is a 3D plot to see variation in char yield with biomass quantity and plastic quantity. When plastic quantity is increased with lower biomass quantity there is a small increment in char yield. Polypropylene plastic is primarily composed of carbon-based polymers. When the PP plastic quantity is increased, the carbon content in the reactor also increases. This higher carbon content contributes to the formation of char during the pyrolysis process. The presence of PP plastic, even in small amounts, provides additional carbon-rich material that can contribute to the increment in char yield. As observed from the ultimate analysis of banana peels and PP, where the carbon content is higher, it can be concluded that as the quantity of both plastic and biomass increases, it will lead to an overall increase in char yield.^44^

With a low banana peel biomass quantity, the available biomass material for pyrolysis is relatively limited. As a result, the remaining material, which includes polypropylene plastic, becomes a major contributor to the char yield. The higher microwave power levels enhance the conversion of the remaining material into char, leading to a significant increase in char yield in microwave co-pyrolysis (Fig. 5(e)). Polypropylene plastic, when exposed to high temperatures, undergoes thermal degradation and carbonization. Increasing the microwave power facilitates the carbonization of the plastic, leading to the formation of char. The presence of polypropylene plastic, even in low quantities, contributes to the overall carbon content available for char formation. The higher microwave power levels enhance the carbonization process and result in a higher char yield.^40^ It can be observed that with high biomass content as microwave power is increased it leads to lower char yield. At higher microwave power levels, the availability of oxygen in the system may become limited, increasing in the carbon gasification process. The high temperature and the presence of volatile compounds promote the reaction between carbon and available oxygen, leading to the formation of carbon dioxide (CO_2_) and carbon monoxide (CO) gases. This gasification process consumes the carbon content, reducing the amount of carbon available for char formation and resulting in a decrease in char yield. With a high biomass quantity, the residence time of the material within the reactor may be relatively short, especially at higher microwave power levels. The limited residence time may not allow sufficient time for complete pyrolysis and char formation. Instead, the biomass undergoes partial decomposition, yielding more volatile compounds and reducing the char yield.

In the absence of a significant PP plastic quantity, the char yield is primarily influenced by the carbon content present in the biomass material. With a low PP plastic quantity, a larger proportion of the carbon content from the biomass contributes to the formation of char. The increased microwave power levels enhance the thermal decomposition of the biomass, resulting in a higher production of char.^45^ With a low PP plastic quantity, the contribution of the plastic material to the overall char formation may be relatively minimal. The absence of a significant PP plastic quantity allows biomass to play a more dominant role in char formation. The increased microwave power levels mainly affect the thermal decomposition of the biomass, leading to an increase in char yield, as observed in Fig. 5(f). It is also observed that with higher plastic content as microwave power is increased from 300 W to 600 W, there is a decrease in char yield.

#### 3.3.3 Gas yield

The *R*^2^ plot for gas yield prediction for microwave-assisted co-pyrolysis of biomass and plastic is shown in Fig. 4(c). The SVR model developed predicted the gas yield with an *R*^2^ value of 0.9408, which gives a high-accuracy result when compared to experimental data. A higher *R*^2^ value implies that the model developed is in line with the experimental data provided to it. The 3D plot for the effect on gas yield with biomass quantity and plastic quantity is shown in Fig. 5(g). It is observed that increasing the plastic quantity with lower biomass leads to a marginal increase in gas yield. Increasing the PP plastic quantity, especially with a lower biomass content, can promote the generation of volatile compounds during pyrolysis. Polypropylene plastic undergoes thermal decomposition, releasing volatile compounds such as hydrocarbons and gases. These volatile compounds contribute to the gas yield in microwave co-pyrolysis. With a higher PP plastic quantity, there is a greater supply of plastic material available for thermal decomposition and volatile compound formation, resulting in a marginal increase in gas yield.^42^

Low microwave power levels may result in longer residence times or insufficient temperatures for complete pyrolysis. Incomplete pyrolysis means that a portion of the biomass and plastic materials remain unconverted, resulting in lower gas yield. With a higher banana peel biomass quantity, the available microwave energy may be further distributed among the increased material, reducing the energy available for each component's efficient pyrolysis. This incomplete pyrolysis can contribute to the observed decrease in gas yield, as shown in Fig. 5(h). Increasing microwave power with low or high amounts of biomass leads to higher gas yield as there is more heating of feedstock at higher power. Similarly, Fig. 5(i) shows that with an increase in microwave power gas yield increases significantly. Higher microwave power levels generate more intense heat within the reactor. This elevated temperature promotes the rapid breakdown of biomass and plastic materials, resulting in a greater release of volatile gases. The increased energy input enables more efficient conversion of the organic compounds into gases, leading to a significant increase in gas yield.^41^

#### 3.3.4 Energy consumption parameters

The heating rate in microwave-assisted co-pyrolysis refers to the rate at which the temperature increases within the reactor during the co-pyrolysis process of banana peel biomass and polypropylene (PP) plastic using microwave energy. The heating rate is an important parameter that affects the overall reaction kinetics and product yields. The amount of energy delivered to the system is determined by the microwave power level. Higher microwave power results in a faster heating rate because the materials absorb more energy, generating a rapid temperature increase. The dielectric characteristics and thermal conductivity of banana peels and PP plastic determine how successfully they absorb microwave energy. Microwave radiation is more efficiently absorbed by materials with greater dielectric constants and lower thermal conductivity, resulting in a faster heating rate. The distribution of microwave energy and, as a result, the heating rate can be influenced by the size and form of the sample. Smaller samples or finely powdered materials often heat up faster than larger or bulkier samples. The heating rate can be affected by the reactor's design and geometry. The size of the reactor and the arrangement of materials within the reactor can all influence how microwave energy is disseminated and absorbed by the biomass and plastic.

In this study, the average heating rate was calculated from the experimental readings and then this data was used to develop an SVR model to predict the average heating rate. It can be observed in Fig. 4(d) that the model developed was able to predict the average heating rate with high accuracy and an *R*^2^ value of 0.98.

The percentage of conversion was calculated to find the amount of initial mass of feedstock converted to oil and gas. The percentage of conversion gives useful information about the pyrolysis process's efficiency and the extent to which starting materials are turned into useable products. Fig. 4(e) shows the *R*^2^ plot for the prediction of the percentage of conversion by using the SVR model. It can be observed that the model predicted the percentage of conversion with a high *R*^2^ value of 0.99. Monitoring and optimizing the percentage of conversion in microwave co-pyrolysis can aid in understanding and improving overall process efficiency, selecting the best conditions for targeted product yields, and assessing the possibility of resource recovery from biomass and plastic waste.

In microwave co-pyrolysis, the rate of mass loss (wt% min^−1^) refers to the rate at which the overall mass of the biomass and plastic components reduces overtime during the pyrolysis process. It is the weight percentage of material lost per unit of time, and it provides information about the kinetics of the co-pyrolysis reaction. The rate of mass loss is frequently used to evaluate and compare the pyrolysis behavior of various biomass-plastic combinations and to optimize process settings for desired results. It assists in comprehending the time-dependent changes in material composition as well as the efficiency of conversion into various products such as gases, liquids, and solids. Insights into the pyrolysis kinetics, comparing different experimental conditions, and assessing the appropriateness of microwave co-pyrolysis as a viable waste-to-energy conversion technology can be found by analyzing the rate of mass loss. Fig. 4(f) gives an *R*^2^ plot of the rate of mass loss predicted by the SVR model with an *R*^2^ value of 0.98.

The susceptor thermal energy has a direct impact on the heating efficiency and temperature distribution within the reactor. By precisely identifying the thermal energy of the susceptor, researchers may optimize its design and features to maximize microwave energy absorption and conversion into thermal energy. This optimization improves heating uniformity, reaction speeds, and control over the pyrolysis process parameters. The susceptor thermal energy has a direct impact on the heating efficiency and temperature distribution within the reactor. By precisely identifying the thermal energy of the susceptor, researchers may optimize its design and features to maximize microwave energy absorption and conversion into thermal energy. This optimization improves heating uniformity, reaction speeds, and control over the pyrolysis process parameters. The determination of the susceptor thermal energy allows for a complete investigation of the heating profile within the reactor. It aids in the detection of temperature gradients, hot patches, and uneven heating. This knowledge can be used to fine-tune the location and distribution of the susceptor material, resulting in more uniform and controlled heating. A greater understanding of the heating profile allows for more exact temperature control, allowing the pyrolysis conditions to be optimized for desired product yields and compositions. The SVR model was developed to predict the susceptor thermal energy (J g^−1^) and it predicted the output with an *R*^2^ value of 0.81 as shown in Fig. 4(g).

### 3.4 Solid product characterization

#### 3.4.1 Fourier transform infrared spectroscopy

**Fig. 6:**
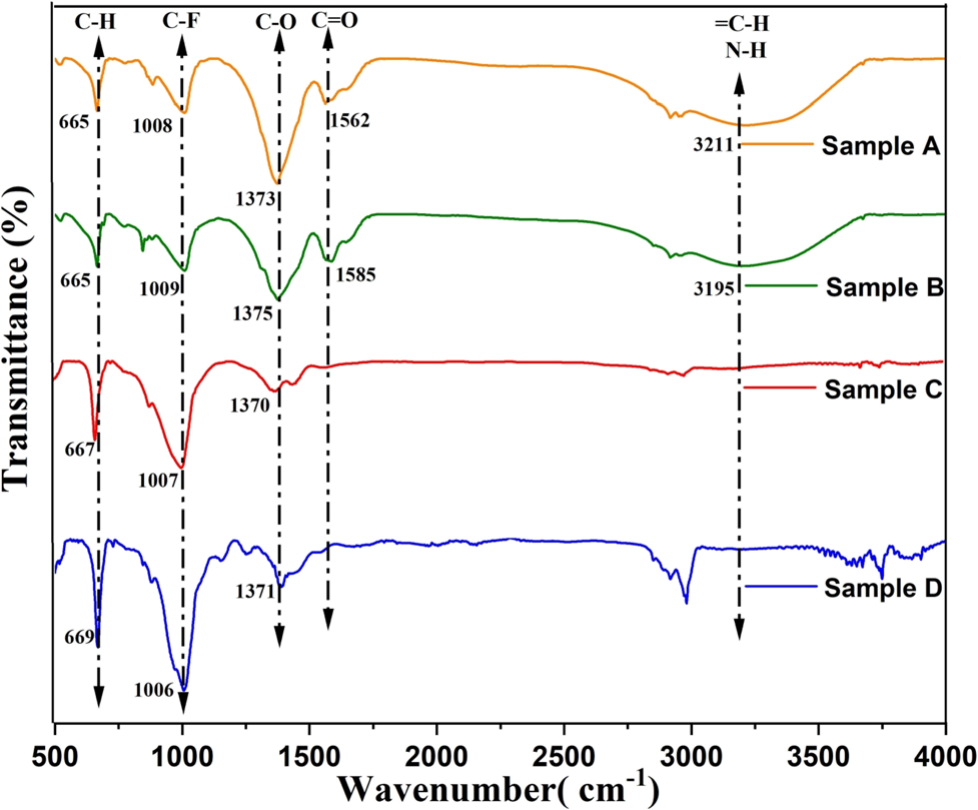
FTIR plots for char obtained from microwave assisted co-pyrolysis of biomass.

Fourier Transform Infrared (FTIR) spectroscopy is a widely used technique to analyze the interaction between infrared radiation and molecular bonds in a sample. When exposed to varying IR wavelengths, molecules absorb specific frequencies, causing vibrational transitions that produce characteristic absorption peaks. These peaks reveal the presence of functional groups, enabling qualitative and semi-quantitative analysis of the sample's chemical composition. In this study, FTIR analysis was conducted on char obtained from microwave-assisted co-pyrolysis of biomass and plastic. Selected samples with the highest char yields were analyzed, and the corresponding FTIR spectra are shown in Fig. 6. It can be observed that there are absorption peaks at wavenumbers like 1006.60 cm^−1^ and 669.02 cm^−1^. This peak corresponds to a specific wavenumber where the char material absorbs infrared light. The fact that the transmittance is relatively high at this wavenumber suggests that there is not much absorption occurring at this point.^46^ This could indicate that there might not be a strong presence of functional groups or chemical bonds that absorb this wavenumber. Based on these peaks, it appears that the char sample contains functional groups associated with C–O stretching vibrations and C–H bending vibrations.^47^ This suggests the presence of oxygen-containing compounds and organic compounds with hydrocarbon chains in the char sample.

#### 3.4.2 X-ray diffraction

X-ray diffraction (XRD) is a powerful technique for analyzing the crystal structure of materials based on the diffraction of X-rays by the regular atomic arrangement in a crystal lattice. By measuring the angles and intensities of diffracted beams, key information on lattice parameters, phase composition, and atomic structure can be obtained. In this study, XRD analysis was performed on char samples with the highest yield from microwave-assisted co-pyrolysis to evaluate their structural properties and phase composition. To quantify the structural features, the crystallite size (*D*) of the graphitic domains was estimated using the Scherrer equation.9
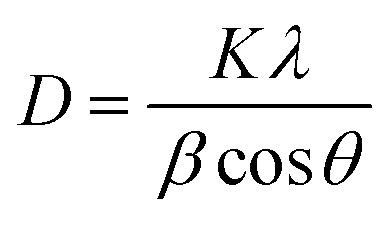
where *K* is the shape factor (0.9), *λ* is the X-ray wavelength (0.15406 nm for Cu Kα radiation), *β* is the full width at half maximum (FWHM) in radians, and *θ* is the Bragg angle.^48^ A crystallinity index can be calculated to assess the degree of structural ordering within the carbon matrix. This detailed analysis provides valuable insights into the phase composition, crystal structure, and thermal behavior of the char, contributing to the understanding of pyrolysis mechanisms and enabling the optimization of process parameters for targeted material properties.

**Fig. 7:**
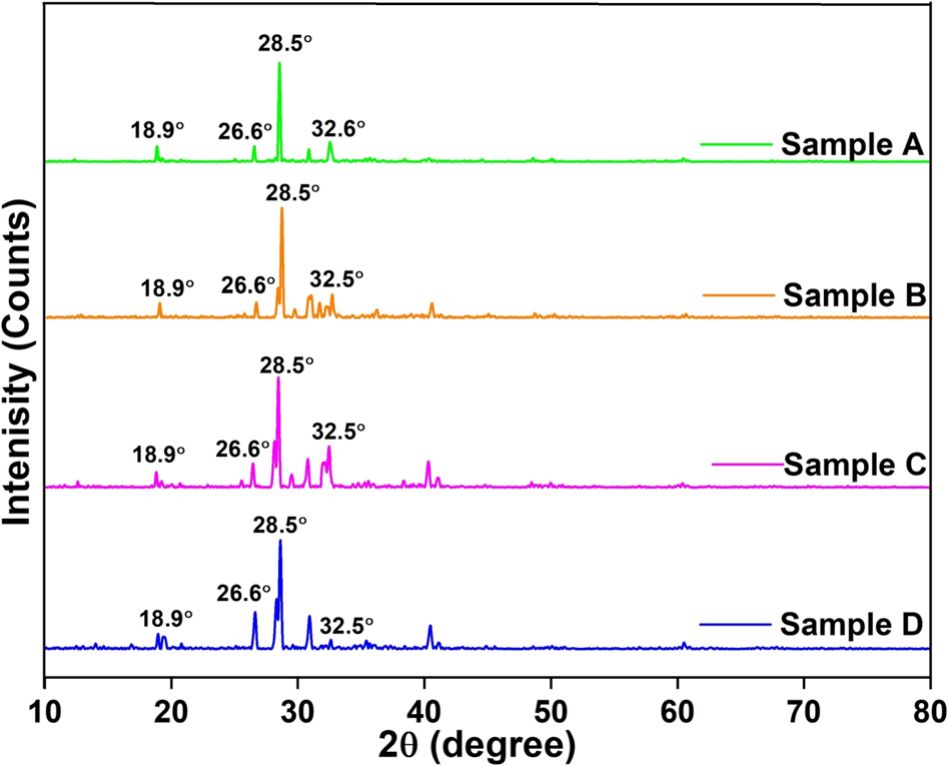
XRD plots for char obtained from microwave-assisted co-pyrolysis of biomass and plastic.

The diffraction patterns, shown in Fig. 7, provide insights into the crystalline changes occurring during pyrolysis and help assess char quality for process optimization.^49^ XRD analysis of char obtained from microwave-assisted co-pyrolysis involves exposing the samples to X-ray radiation and examining the resulting diffraction patterns. The presence of sharp peaks in the XRD spectra indicates the existence of crystalline phases within the char structure.^50^ These crystalline phases could be mineral residues from the original biomass or other compounds that have undergone crystallization during the co-pyrolysis process. The sharpness of the peaks indicates that the atoms or molecules within these crystalline phases are arranged in a highly ordered and repetitive manner.^51^ As observed from Fig. 7 that prominent peaks were observed at 2*θ* = 26.6°, 28.5°, and 32.5°, and these were identified using standard reference patterns from the Joint Committee on Powder Diffraction Standards (JCPDS) database.^52^ Specifically, the peak at 26.6° corresponds to the (002) plane of graphitic carbon (JCPDS no. 41-1487), indicating the development of ordered, turbostratic graphite-like structures. The peak at 28.5° is attributed to calcite (CaCO_3_) (JCPDS no. 05-0586), while the peak at 32.5° corresponds to potassium carbonate (K_2_CO_3_) (JCPDS no. 01-077-1086).^53^ These mineral phases likely originated from the inorganic constituents of the biomass or formed through thermally induced reactions during the pyrolysis process.

#### 3.4.3 Brunauer–Emmett–Teller

Brunauer–Emmett–Teller (BET) analysis is a widely used technique for determining the specific surface area and porosity of solid materials, including char derived from co-pyrolysis. It is based on the adsorption of gas molecules, typically nitrogen, onto the surface of the material at varying relative pressures. By applying the BET equation, the total surface area, including both external and internal surfaces, can be quantified in m^2^ g^−1^. This parameter is critical for evaluating the performance of porous materials in applications such as adsorption, catalysis, and material design, and is extensively used in materials science and related fields.

The BET equation describes this adsorption process:10
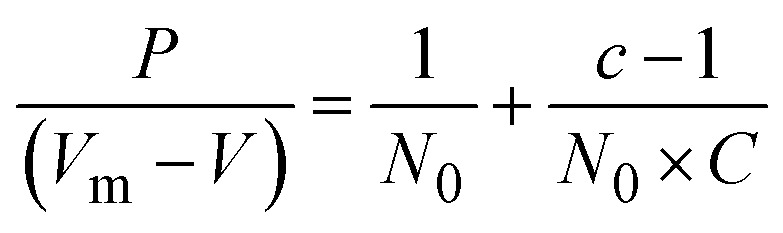
where *P* is the equilibrium pressure of the adsorbate (nitrogen gas), *V*_m_ is the molar volume of the adsorbate, *V* is the volume of gas adsorbed at equilibrium, *N*_0_ is the number of moles of gas required to form a complete monolayer, *C* is the BET constant, *c* is the ratio of the amount of gas adsorbed on the surface at a particular pressure to the amount adsorbed in a complete monolayer.

BET surface area analysis of char produced from microwave-assisted co-pyrolysis of biomass and plastic was conducted using the Autosorb iQ Station 1 analyzer. The results of the multipoint BET analysis for the sample with the highest char yield are given in Table 3. The nitrogen adsorption isotherm exhibits a gradual increase in adsorbed volume at low relative pressures, indicative of monolayer formation. Specifically, monolayer adsorption is observed at a relative pressure (*P*/*P*_o_) of 0.0505, corresponding to an adsorbed volume of 0.2592 cm^3^ g^−1^. As the relative pressure increases, the adsorption curve shows a steeper increase, suggesting multilayer adsorption behavior. Across the measured range of relative pressures (0.005 to 0.3), the adsorbed volume increases from 0.25 cm^3^ g^−1^ to 1.74 cm^3^ g^−1^, highlighting the porous nature of the char and its capacity for multilayer adsorption. These results provide key insights into the surface characteristics of the char, which are important for evaluating its suitability in adsorption and catalytic applications.

**Table 3:** BET analysis char obtained from microwave-assisted co-pyrolysis of biomass and plastic

Relative pressure (*P*/*P*_0_)	Volume @STP cm^3^ g^−1^	1/[*W*((*P*_o_/*P*) − 1)] 1 g^−1^
5.04672 × 10^−2^	0.2592	1.6407 × 10^2^
1.12661 × 10^−1^	0.6257	1.6235 × 10^2^
1.75537 × 10^−1^	0.9078	1.8765 × 10^2^
2.37866 × 10^−1^	1.3838	1.8047 × 10^2^
3.00196 × 10^−1^	1.7407	1.9178 × 10^2^

## 4 Conclusion

In this study, 13 experiments were performed for microwave-assisted co-pyrolysis of biomass and plastic with banana peel powder (0.2–1 mm) as biomass and polypropylene as plastic. Design of experiments was performed in which biomass quantity, plastic quantity, and microwave power were varied as 5 g, 10 g, 15 g, and 300 W, 450 W, 600 W, respectively. Oil yield, char yield, gas yield, and heating rate were noted after every run of experiments for 10 minutes. After the collection of data, the percentage of conversion, the rate of mass loss, and the susceptor thermal energy were calculated. Further, this data was used to train the SVR model and predict the outputs using biomass quantity, plastic quantity, and microwave power as input variables. The SVR model was able to predict the outputs with higher accuracy, with an *R*^2^ value between 0.81–0.99. 3D plots were generated to see the effect of input variables on product yields. Further product analysis of char obtained from microwave-assisted co-pyrolysis was done using FTIR. XRD and BET. FTIR analysis shows the presence of functional groups associated with C–O stretching vibrations and C–H bending vibrations. XRD of char shows sharp peaks, suggesting that the char sample contains crystalline phases. BET of char shows how multilayer adsorption takes place as the relative pressure is increased. TGA of banana peel biomass shows the presence of moisture content and thermal degradation of cellulose. Also, the TGA of polypropylene plastic shows how polymer chains break down into smaller fragments, resulting in the release of volatile products, such as hydrocarbons and gases, which are responsible for the mass loss. Key challenges are associated with scaling up microwave-assisted co-pyrolysis, including achieving uniform microwave heating in larger reactor volumes, managing increased energy demands, and ensuring catalyst stability and reusability over extended cycles.

## Author contributions

S. Rajpurohit: conceptualization, methodology, visualization, investigation, writing – original draft. Shruti Sinha: formal analysis, investigation, writing – original draft, validation. Ramesh Potnuri: formal analysis, investigation, writing – original draft, validation. Harshini Dasari: supervision, conceptualization, funding acquisition, resources, writing – review & editing. Chinta Sankar Rao: supervision, conceptualization, funding acquisition, resources, writing – review & editing.

## Conflicts of interest

The authors declare that they have no known competing financial interests or personal relationships that could appear to influence the work reported in this paper.

## Supplementary Material

RA-015-D5RA03913D-s001

## Data Availability

The authors confirm that the data supporting the findings of this study are available within the article [and/or] its SI. Supplementary information is available. See DOI: https://doi.org/10.1039/d5ra03913d.
