# Supplementary material for: Microwave assisted catalytic co-pyrolysis of banana peels and polypropylene: experimentation and machine learning optimization
Source: RSC Adv. 2025 Aug 11;15(35):28325–37. doi: 10.1039/d5ra03913d (PMC12376775; doi:10.1039/d5ra03913d)
Supplement: RA-015-D5RA03913D-s001 [file RA-015-D5RA03913D-s001.pdf]

Supplementary Information (SI) for RSC Advances.
This journal is © The Royal Society of Chemistry 2025

Microwave Assisted Catalytic Co-Pyrolysis of Banana Peels and Polypropylene: Experimentation and Machine Learning Optimization

Nilesh S. Rajpurohit[a], Shruti Sinha[a], Ramesh Potnuri[a], Chinta Sankar Rao[a*], Harshini Dasari[b*]

[a] Control Systems & Machine Learning Research Laboratory, Department of Chemical Engineering, National Institute of Technology Karnataka, Surathkal - 575025, India

[b]Chemical Engineering Department, Manipal Institute of Technology Manipal Academy of Higher Education, Manipal, 576104, Udupi, Karnataka, India

Email of Corresponding authors: csrao@nitk.edu.in (C. Sankar Rao)

harshini.dasari@manipal.edu (Dr. Harshini Dasari)

Supplementary Material

Support Vector Regression

Consider a training dataset comprising ***N*** samples, where each individual sample is defined by an input vector $\boldsymbol{x_i}$ and a corresponding target output $\boldsymbol{y_i}$. The primary objective of SVR is to determine a function ***f(x)*** that can effectively map the input ***x*** to the predicted output ***y***, capturing the underlying relationship between the variables with minimal prediction error.

The SVR model's fundamental formula is given by:

$$f(x) = w^T \times x + b \quad \textbf{(7)}$$

where ***w*** is the weight vector, and ***b*** is the bias term. The weight vector w determines the hyperplane's orientation, and its position is changed by the bias term ***b***. SVR uses a kernel function to implicitly transfer the input variables to a higher-dimensional space to capture non-linear correlations. The selection of the kernel function is crucial and is based on the issue at hand. The linear, polynomial, radial basis function (RBF), and sigmoid are frequently used kernel functions.

The optimization problem for SVR involves minimizing the following objective function:

$$minimize \quad \frac{1}{2}||w||^2 + C\sum\left(\xi_i + \xi_i^*\right) \quad (8)$$

Subject to: $\quad y_i - < w,x_i > - b \leq \varepsilon + \xi_i$

$$< w,x_i > + b - y_i \leq \varepsilon + \xi_i^*$$

$$\xi_i, \xi_i^* \geq 0$$

In this formulation, $||\mathbf{w}||^2$ represents the squared Euclidean norm of the weight vector, while C is the regularization parameter that regulates the trade-off between model complexity and the generalization error. Fitting errors, ξ_i and ξ_i^* are slack variables that allow for deviations from the margin, and ε is the width of the epsilon tube [1].

The dual optimization issue for the SVR model must also be solved to get a set of Lagrange multipliers. The support vectors, which are essential to the final model, are chosen using these multipliers. The SVR model can be used to forecast future data once it has been trained on experimental data. The learned weight vector w and bias term b are used by the model to compute the output given a fresh set of input variables [2].

The benefit of using SVR to analyze the experimental data from microwave co-pyrolysis is its capacity to handle non-linear correlations and capture intricate connections between input and output variables. SVR can successfully simulate the co-pyrolysis process and estimate product yields by carefully choosing the kernel function and optimizing the hyperparameters. Additionally, SVR enables researchers to pinpoint the most crucial variables influencing the co-pyrolysis results by providing insight into the impact of various input variables on the output.

For hyperparameter tuning, the GridSearchCV method was used. The GridSearchCV method is commonly employed for hyperparameter tuning of SVR models. Hyperparameters are settings that are established before training the model and are not learned from data. The GridSearchCV method does an exhaustive search across a predefined grid of hyperparameters to determine the optimum combination [3]. The K-fold cross-validation technique is frequently used to test the performance of different hyperparameter combinations. The dataset is separated into K

equal-sized subsets or folds using this technique. The SVR model is trained using K-1 folds, while the remaining fold is validated. This procedure is done K times, with each fold serving as the validation set just once.

The GridSearchCV method starts by creating a grid of hyperparameters and their values to be investigated. The regularization parameter (C), the epsilon-tube parameter (epsilon), and the kernel type are examples of hyperparameters [3]. Following that, an SVR model with the supplied hyperparameters is constructed. The SVR model, the parameter grid, the desired performance metric, and the number of cross-validation folds (K) are then used to create a GridSearchCV object. The GridSearchCV object is fitted to the training data by iterating through all possible grid hyperparameter combinations. An SVR model is trained and evaluated for each combination using K-fold cross-validation [4]. The GridSearchCV method returns the results for each hyperparameter combination, calculating the average performance metric (e.g., mean squared error) across all K folds. The best model, as decided by the specified performance metric, can be obtained via the GridSearchCV object's ‘best estimator’ attributes. Other properties, such as ‘best params’, reveal the particular hyperparameter values for the best model. Researchers may effectively tune the hyperparameters of SVR models using GridSearchCV and K-fold cross-validation. This method automates the process of determining the best hyperparameter combination by exhaustively analyzing its impact on model performance using cross-validation. The ultimate result is a fine-tuned SVR model with increased generalization to new data [5].

Table S1. Performance of the models for various target variables

Metrics	Oil yield	Gas yield	Char yield	HR	Conv.	Mass loss	Susceptor thermal energy
R^2	0.9999	0.9408	0.9990	0.9861	0.9990	0.9787	0.8104
MAE	0.1000	1.5748	0.2603	0.4630	0.2603	0.1611	30.2704
MAPE	0.0036	0.0398	0.0087	0.0080	0.0040	0.0267	0.0404
RMSE	0.1000	2.7759	0.5078	1.1827	0.5078	0.2355	78.6182

*HR: Heating rate; Conv.: Conversion

[1] S. Li, K. Xu, G. Xue, J. Liu, Z. Xu, Prediction of coal spontaneous combustion temperature based on improved grey wolf optimizer algorithm and support vector regression, Fuel 324 (2022) 124670. https://doi.org/10.1016/j.fuel.2022.124670.

[2] A. Mohammadian, Z. Mortezaei, Y. Nejatyjahromy, Informatics in Medicine Unlocked Fast rank-based normalization of miRNA qPCR arrays using support vector regression, Informatics Med. Unlocked 39 (2023) 101265. https://doi.org/10.1016/j.imu.2023.101265.

[3] B. Aljaman, U. Ahmed, U. Zahid, V.M. Reddy, S.M. Sarathy, A. Gani, A. Jameel, A comprehensive neural network model for predicting flash point of oxygenated fuels using a functional group approach, Fuel 317 (2022) 123428. https://doi.org/10.1016/j.fuel.2022.123428.

[4] S. Uddin, Z. Hossain, S. Rahman, S.K. Sarker, On the protection of power system : Transmission line fault analysis based on an optimal machine learning approach, Energy Reports 8 (2022) 10168–10182. https://doi.org/10.1016/j.egyr.2022.07.163.

[5] D.L. Zou, L.L. Wu, Y.F. Hao, L. Xu, J.J. Chen, Composition-strength relationship study of ultrahigh performance fiber reinforced concrete (UHPFRC) using an interpretable data-driven approach, 392 (2023). https://doi.org/10.1016/j.conbuildmat.2023.131973.
